# Supplementary material for: The flavonoid corylin exhibits lifespan extension properties in mouse
Source: Nat Commun. 2022 Mar 9;13:1238. doi: 10.1038/s41467-022-28908-2 (PMC8907184; doi:10.1038/s41467-022-28908-2)
Supplement: Supplementary file 1 — Supplementary Information [file 41467_2022_28908_MOESM1_ESM.pdf]

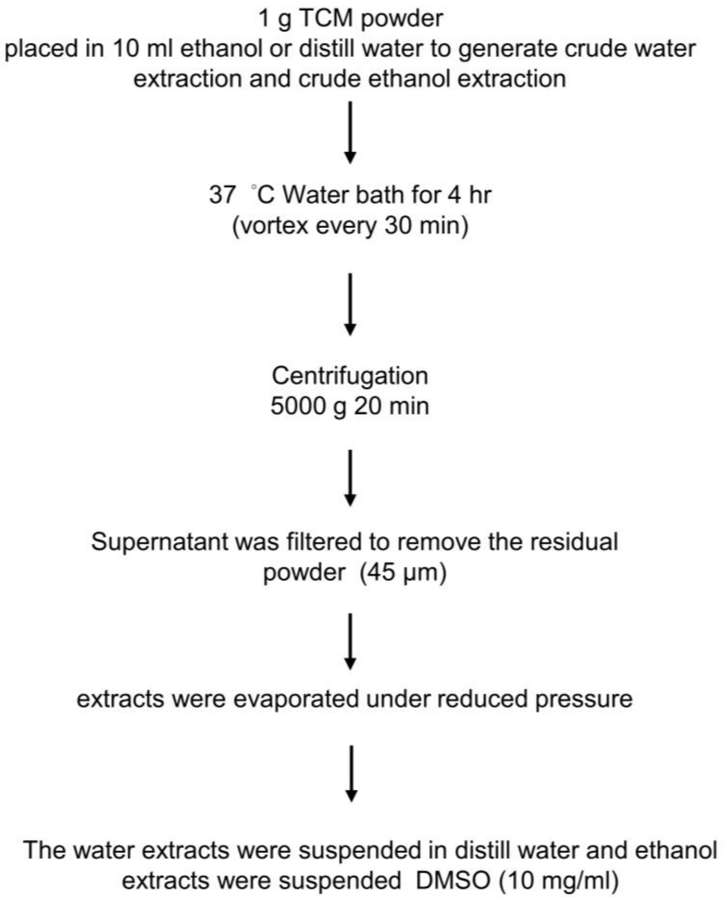

Supplementary Figure 1 The standard protocol for TCM formula extraction. To generate the TCM extract, 1 g of 33 types of TCM and 6 types of herbal formula powder were placed in 10 ml of ethanol or distilled water, respectively. Next, each sample was placed in a 37°C water bath for 4 h and vortexed every 30 min to ensure complete extraction. After the extraction procedures, samples were pelleted by centrifugation at 5000 g for 20 min to remove the undissolved powder. To acquire the pure concentrate without undissolved powder, each extract was filtered through a 45 µm syringe filter and concentrated using a rotary evaporator. Before the screening and validation procedure, samples were weighed and dissolved in a 10 mg/ml solution (water extracts were dissolved in distilled water, and the ethanol extracts were dissolved in DMSO).

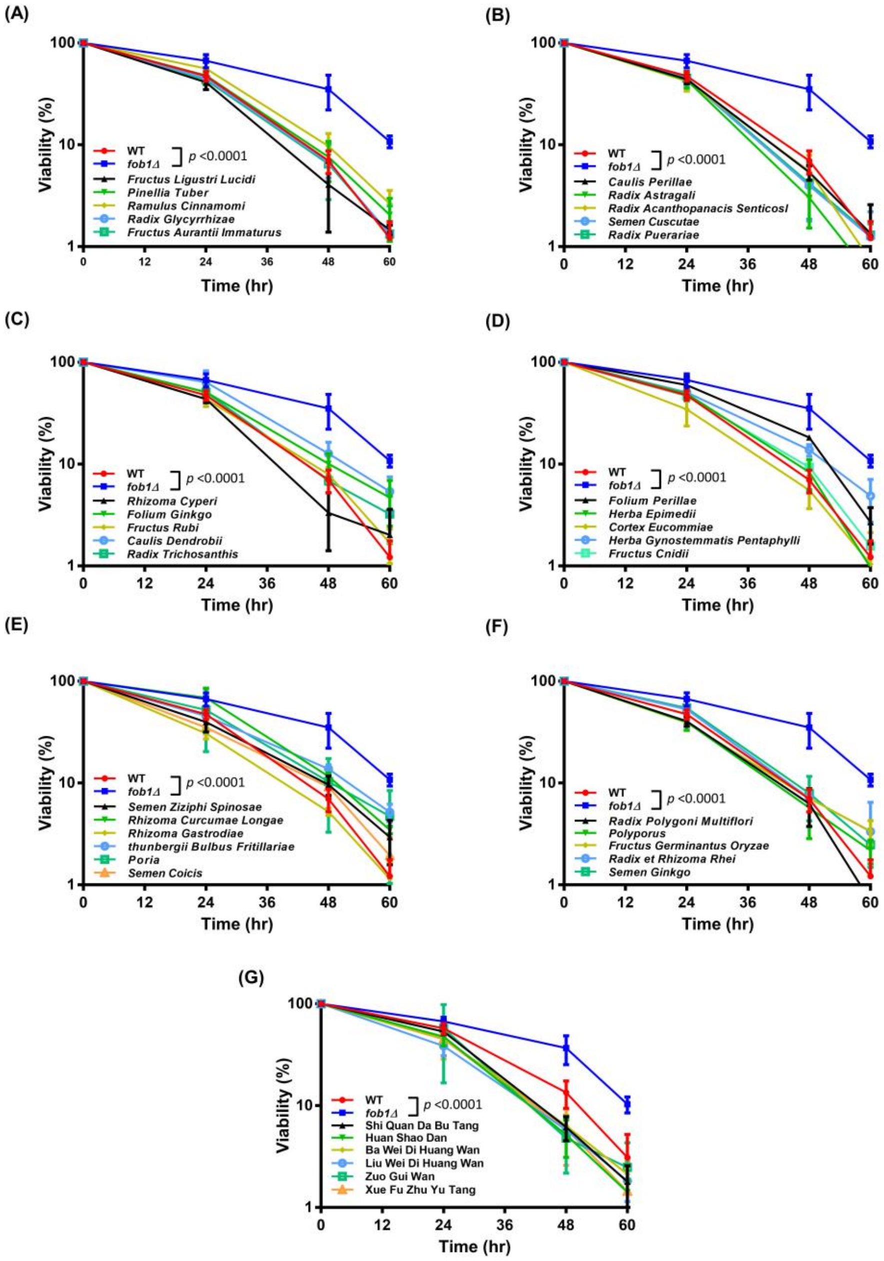

Supplementary Figure 2 Validation of the RLS extension candidate from ethanol extraction of single and herbal formula TCMs. Viability curves of the haploid MEP strain ZHY1 in liquid YEPD containing 10 μg/ml of extract from (A-F) 31 different types of single herbs and (G) 6 different types of herbal formulas. Cultures were incubated at 30°C for 60 h. The viability is presented as CFUs per 500 μl, and this value was determined by harvesting samples at the indicated time points. Data are represented as mean ± SD from 3 biologically independent experiments. *p* values were determined by two-way ANOVA followed multiple comparisons test.

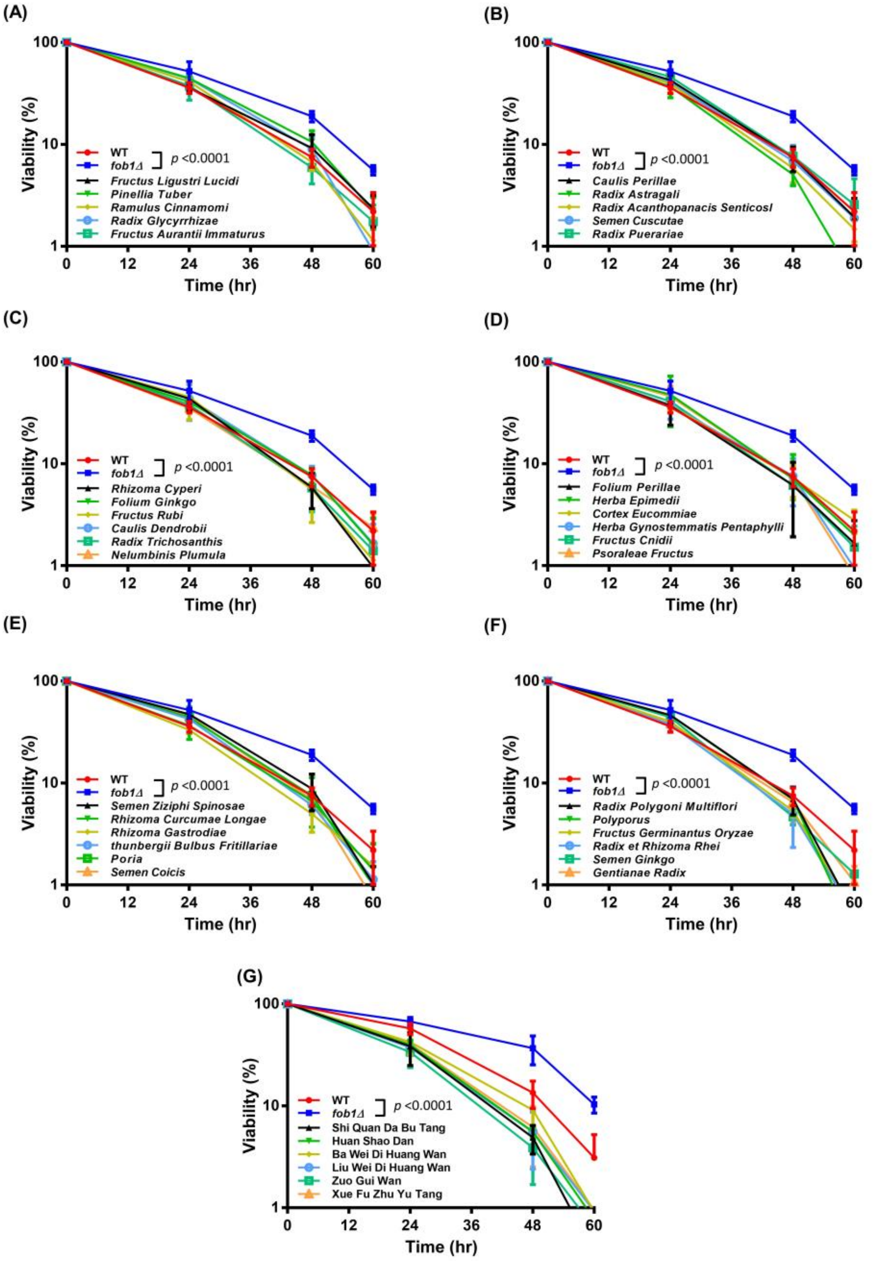

Supplementary Figure 3 Validation of the RLS extension candidate from water extraction of single and herbal formula TCMs. Viability curve of the haploid MEP strain ZHY1 in liquid YEPD containing 10 μg/ml of extract from (A-F) 34 different types of single herbs and (G) 6 different types of herbal formulas. Cultures were incubated at 30°C for 60 h. The viability is presented as CFUs per 500 μl, and this value was determined by harvesting samples at the indicated time points. Data are represented as mean ± SD from 3 biologically independent experiments. *p* values were determined by two-way ANOVA followed multiple comparisons test.

(A)

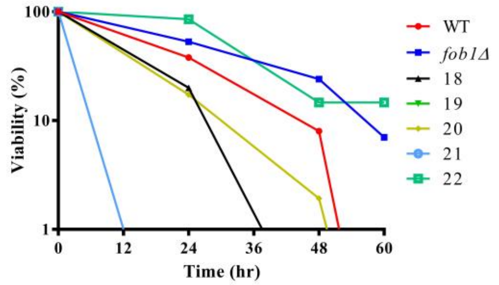

(B)

CFUs of the indicated time points (hr)				
	0	24	48	60
WT	246	94	20	1
fob1Δ	253	133	61	18
18	5	1	0	0
19	0	0	0	0
20	52	9	1	0
21	63	0	0	0
22	41	35	6	6

Supplementary Figure 4 MEP assay of chalcones from the hexane extract of *Psoralea corylifolia* Visualization curve of the haploid MEP strain ZHY1 in liquid YEPD containing (A) 15 μM chalcones: (18) 3”-hydroxy-isopentyl-isoliquiritigenin, (19) isobavachromene, (20) psorachromene, (21) 4-methoxybavachalcone, and (22) isobavachalcone. Cultures were incubated at 30°C for 60 h. (B) The table demonstrates the cytotoxicity of 15 μM chalcones at time point 0. The viability is presented as CFUs per 500 μl, and this value was determined by harvesting samples at the indicated time points.

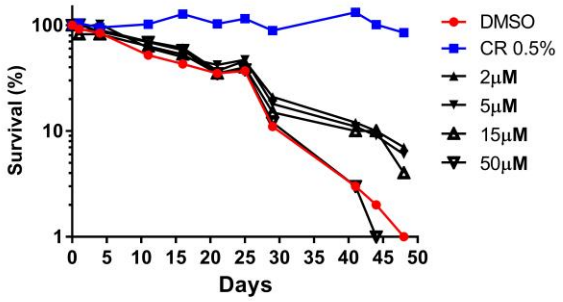

Supplementary Figure 5 Chronological lifespan (CLS) survival curves of BY4741 in SC media with or without corylin treatment. The CLS experiment was conducted on a BY4741 background and carried out in 2% glucose SC media. Cells were grown overnight at 30°C in SC medium and then diluted 1:5 in a 125 ml flask with the indicated treatment at a final volume of 20 ml at 30°C and 150 rpm (CR group, glucose reduced to 0.5%). When cultures reached saturation in SC medium (48 h), aliquots from the culture were harvested, subjected to serial dilution and placed on YEPD plates to form colonies; this time point was considered day 0. The viability was determined by the number of CFUs at each indicated time point compared to that at day 0.

(A)

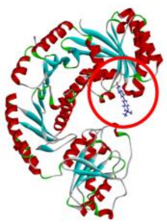

Pose	CDOCKER_INTERACTION_ENERGY
1	-28.8055
2	-30.0823
3	-28.5135
4	-28.0801
5	-27.9982
6	-27.4801
7	-27.4719
8	-26.8428
9	-26.4607
10	-26.1113

(B)

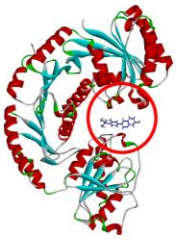

Pose	CDOCKER_INTERACTION_ENERGY
1	-24.768
2	-24.854
3	-25.0767
4	-24.5779
5	-25.0113
6	-24.5772
7	-24.1039
8	-24.6923
9	-23.6507
10	-22.9293

(C)

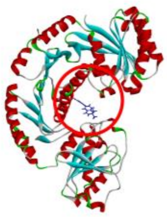

Pose	CDOCKER_INTERACTION_ENERGY
1	-22.238
2	-21.1573
3	-21.0634
4	-21.1252
5	-21.3679
6	-20.8293
7	-20.7731
8	-20.4759
9	-20.0238
10	-20.6798

Supplementary Figure 6 The comparison of 3 different domains that may interact with corylin by the docking analysis. (A-C) The CDOCKER-interaction-energy and superpositions of corylin with Gtr1 (PDB ID: 3R7W). The interaction domains of (A) which contain Ile166 and Trp167 showed stronger interaction with corylin.

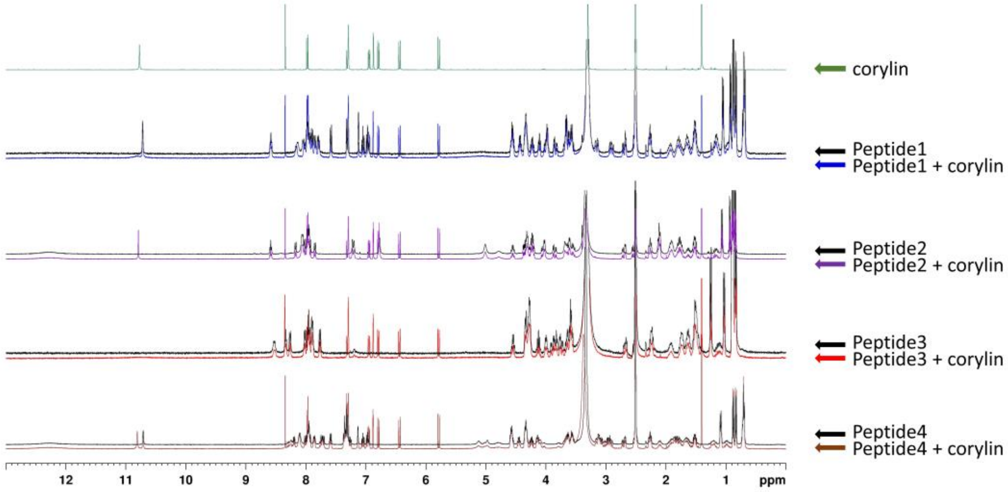

Supplementary Figure 7 Overlaid view of the ^{1}H NMR spectra of the binding experiment recorded at 400 MHz in DMSO-d$_6$.

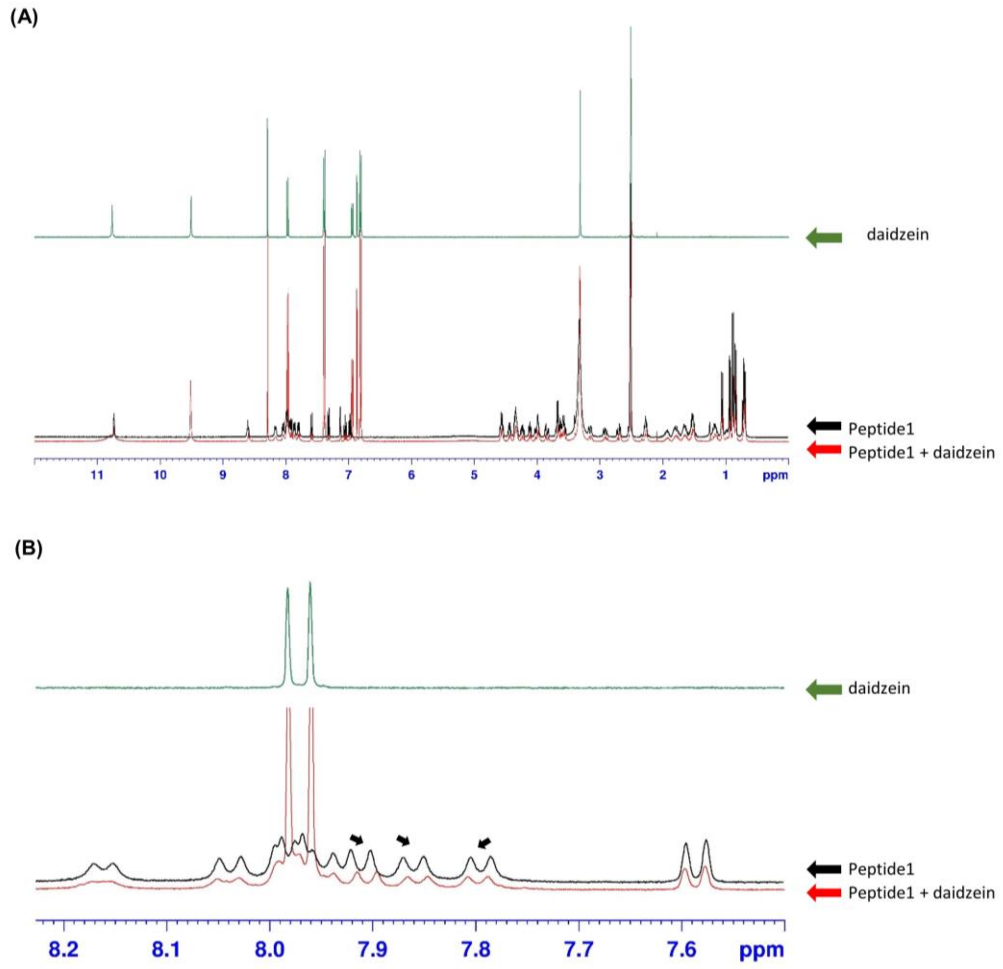

Supplementary Figure 8 ^{1}H NMR spectrum of peptide in the presence or absence of daidzein. a Overlaid view of the ^{1}H NMR spectra of the binding experiment recorded at 400 MHz in DMSO-d_6. **b** Partial ^{1}H NMR spectra. The arrows indicate the upfield chemical shift with the addition of daidzein and with a molar ratio of corylin and peptide of 1:1.

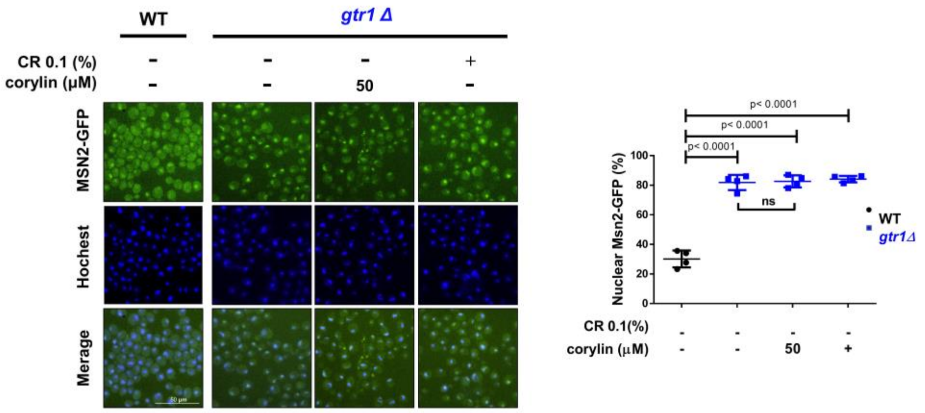

Supplementary Figure 9 Quantification of *gtr1Δ*-MSN2-GFP cells was performed by immunofluorescence microscopy. MSN2-GFP(WT) and *gtr1Δ*-MSN2-GFP (*gtr1Δ*) were inoculated into culture liquid in a microtube for 15 h in a rolling drum. After pregrowth to log phase in 2% glucose, the *gtr1Δ* strain was treated with or without corylin for 1 h, and CR was performed by reducing the glucose to 0.1%. Quantification of MSN2 cells was performed by immunofluorescence microscopy, and the live cells were identified by staining with Hoechst #33342. Data are represented as mean ± SD. *p* values were determined by two-tailed Student's t-test (Each dot represents one biologically independent experiment).

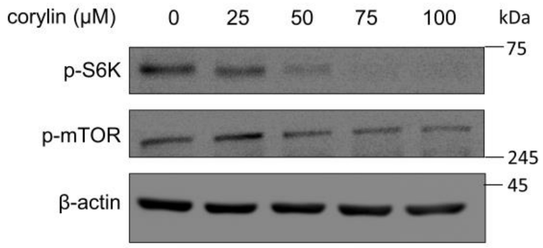

Supplementary Figure 10 Corylin inhibits mTOR1 singling in a dose-dependent manner. U2OS cells were treated corylin with indicated concentrations for 8 hours, then cells were harvested. Cell lysates were conducted immunoblotting to evaluate p-mTOR (S2448), p-S6k(T389), and β-actin expression level. At least three independent replicates were performed for the experiment.

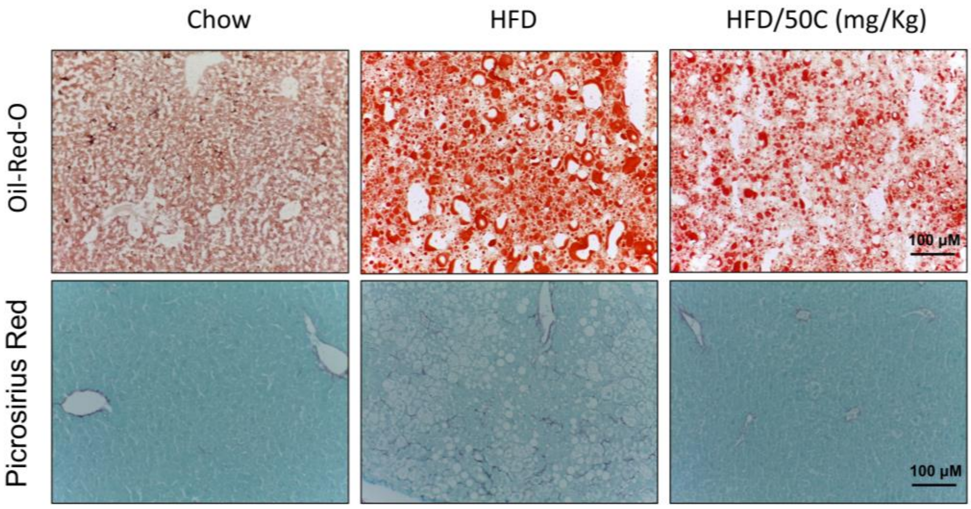

Supplementary Figure 11 Corylin ameliorates HFD-induced hepatic steatosis. Seven-week-old male C57BL/6 mice were divided into three groups: group I (chow), fed a normal chow diet with vehicle orally for 9 weeks; group II, fed a HFD with vehicle orally for 9 weeks (60% high-fat diet, HFD); and group III, fed a HFD with corylin (50 mg/kg/day) orally for 9 weeks. At the end of treatment, liver samples were collected from individual mice. Liver sections were stained with Oil Red O and Picrosirius Red to evaluate accumulation of hepatic lipids and fibrosis, respectively. At least three independent samples were performed for the experiment.

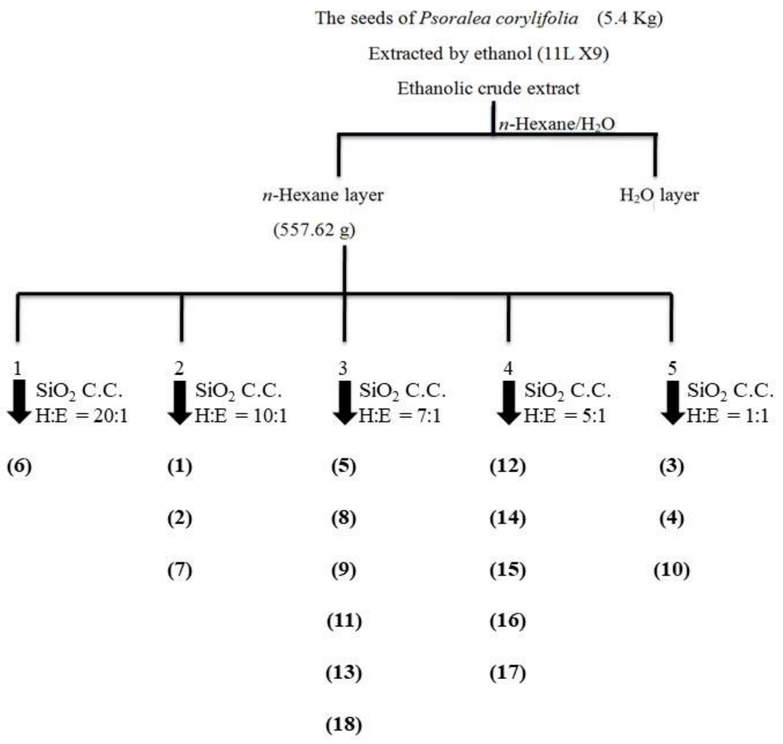

Supplementary Figure 12 Extraction scheme of *P. corylifolia.* The structure of the compounds was presented in figure 2.

(A)

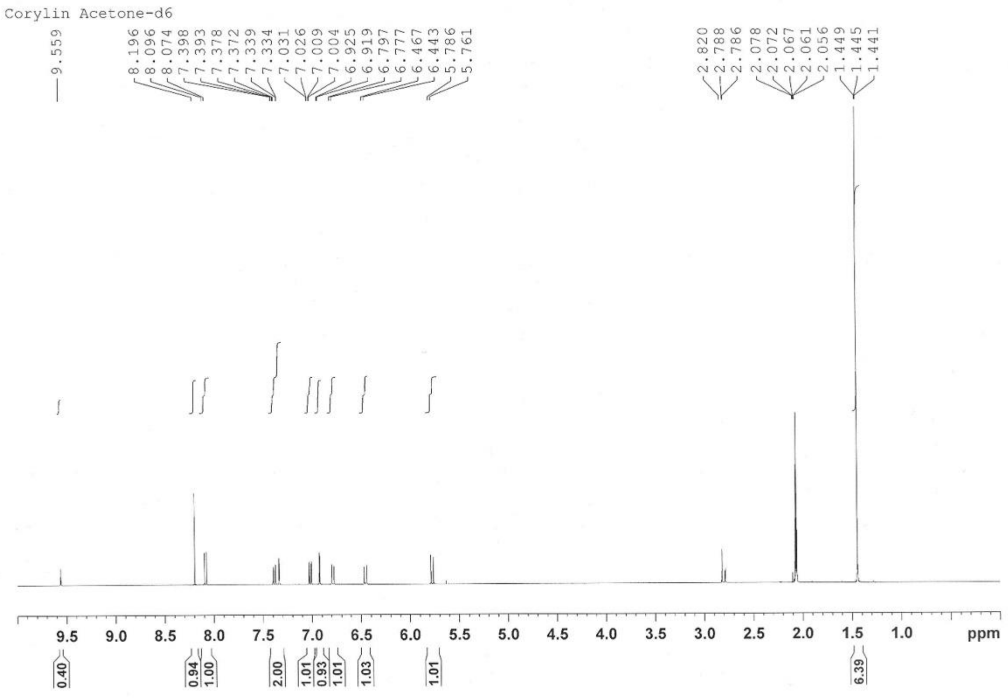

(B)

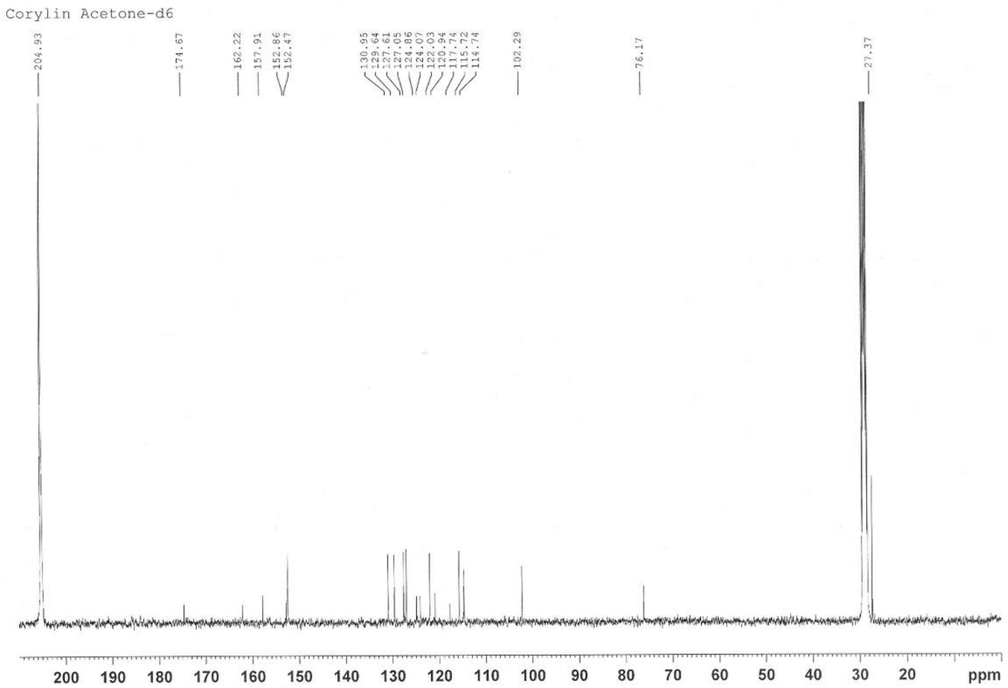

Supplementary Figure 13 The NMR proton spectra of coylin. a ^{1}H NMR Spectrum **b** ^{13}C NMR Spectrum. Corylin**:** White power；Formula: $C_{20}H_{16}O_4$；MP : 245~255°C；UV λ_{max}(MeOH) nm: 305, 247；IR ν_{max}(KBr) cm^{-1}: 3235, 2927, 1625, 1495, 1375, 1274, 1184 ESI-MS *m/z*: 321[M+1]$^{+}$, 343 [M+Na]$^{+}$, 359 [M+K]$^{+}$ ；^{1}H-NMR (Acetone-d_6,

400MHz) δ_H:9.62 (1H, *s*, OH-7), 8.19 (1H, *s*, H-2), 8.06 (1H, *d*, *J* = 8.8 Hz, H-5), 7.38 (1H, *dd*, *J* = 8.0, 2.0Hz, H-6'), 7.32 (1H, *s*, H-2'), 7.01 (1H, *dd*, *J* = 8.8, 2.0 Hz, H-6), 6.91 (1H, *d*, *J* = 2.0 Hz, H-8), 6.77 (1H, *d*, *J* = 8.0 Hz, H-5'), 6.44 (1H, *d, J* = 10.0 Hz, H-1''), 5.77 (1H, *d, J* = 10.0 Hz, H-2''), 1.43 (6H, *s*, CH_3, H-4'',5'') ；^{13}C-NMR (Acetone-d_6, 100MHz) δ_C:175.1 (C=O), 162.7 (C-7), 158.2 (C-9), 153.2 (C-4'), 152.9 (C-2), 131.3 (C-3''), 130.1 (C-6'), 128.0 (C-5), 127.5 (C-2'), 125.3 (C-1'), 124.4 (C-3), 122.4 (C-4''), 121.3 (C-3'), 118.1 (C-10), 116.1 (C-5'), 115.1 (C-6), 102.7 (C-8), 76.6 (C-2''), 27.8 (C-5'', 6'')

Figure		Strain	n	Mean	Median	vs strain i	vs strain ii	vs strain iii	vs strain iv
	i	WT	59	24.7	24				
Fig 1b	ii	*fob1*	60	30.7	31	*p*=0.001			
	iii	ethanol extract of *P. Psoraleae*	59	31.2	34	*p*=0.0002			
	i	WT	104	23.7	24				
Fig 1d	ii	*fob1*	78	30.8	32	*p*<0.0001			
	iii	n-HEAXEN 10 mg/ml	96	29	29	*p*=0.0004			
	i	WT	104	23.7	24				
Fig 1e	ii	*sir2 fob1*	102	22.9	24				
	iii	*sir2 fob1* + n-HEAXEN 10 mg/ml	102	25.5	26.5		*p*=0.0102		
	i	WT	104	23.7	24				
Fig 1f	ii	*tor1*	104	27.5	28	*p*=0.0026			
	iii	*tor1* + n-HEAXEN 10 mg/ml	103	26.9	27		ns		
	i	WT (26.4)	65	26.4	25				
Fig 4a	ii	*fob1* (30.5)	43	31.4	33.5	*p*=0.0096			
	iii	corylin (31)	66	31	33	*p*=0.0046			
	i	WT	72	24.6	23				
Fig 4b	ii	corylin	78	30.9	32.5	*p*<0.0001			
	iii	*sir2 fob1*	78	24.6	25.5				
	iv	*sir2 fob1* + n-HEAXEN 10 mg/ml	75	28.4	29			*p*=0.0316	
	i	WT	97	25	25				
Fig 4c	ii	corylin	77	29.4	31	*p*=0.0023			
	iii	*tor1*	104	27.8	27	*p*=0.0492			
	iv	*tor1* + corylin	100	27.9	29		ns		
	i	WT	102	25	26				
Fig 4i	ii	corylin	101	29	30	*p*=0.0002			
	iii	CR	104	29.2	30	*p*=0.0002			
	iv	CR+corylin	99	28.5	30		ns		
	i	WT	77	24.3	25				
Fig 5f	ii	corylin	75	29.7	31	*p*=0.0001			
	iii	*gtr1*	78	29.5	31	*p*=0.0005			
	iv	*gtr1+corylin*	78	29.5	30		ns		
	i	WT	26	25	26.5				
	ii	corylin (30μM)	26	31.6	32.5	*p*=0.0016			
	iii	corylin (60μM)	26	30.1	33	*p*=0.0044			
Fig 5g	iv	*gtr1*	26	29.8	31.5	*p*=0.0207			
	v	*gtr1*+corylin (30μM)	26	29.3	33.5		ns		ns
	vi	*gtr1*+corylin (60μM)	26	29.7	31.5			ns	ns
	i	WT	51	25.1	27				
Fig 5i	ii	corylin	52	30.8	31.5	*p*=0.0002			
YEPD	iii	pGAL-GTR1	52	28.2	30.5	*p*=0.037			
	iv	pGAL-GTR1 +corylin	51	27.8	30		ns	ns	
	i	WT	47	26	27				
Fig 5j	ii	corylin	46	29.9	32	p=0.0467			
YEPG	iii	pGAL-GTR1	51	24.7	25	ns	*p*=0.0046		
	iv	pGAL-GTR1 +corylin	51	25.2	26	ns		ns	

Supplementary Figure 14 Statistical analysis of RLS experiments were determined by the Gehan-Breslow-Wilcoxon test and deletion mutants were derived from BY4741.

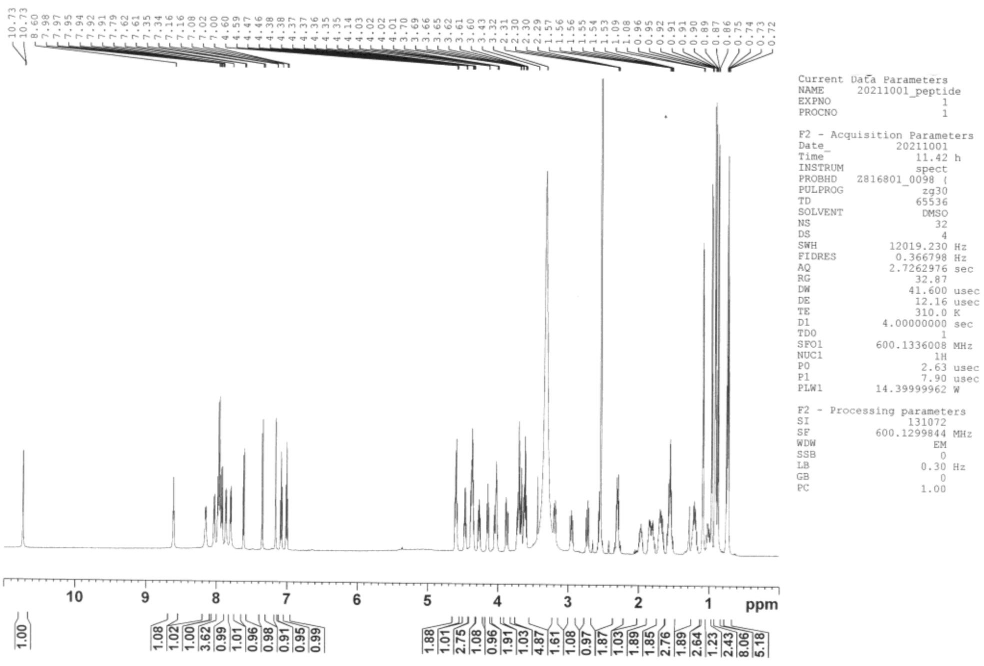

Supplementary Figure 15-1 ^{1}H NMR proton spectrum of peptide 1.

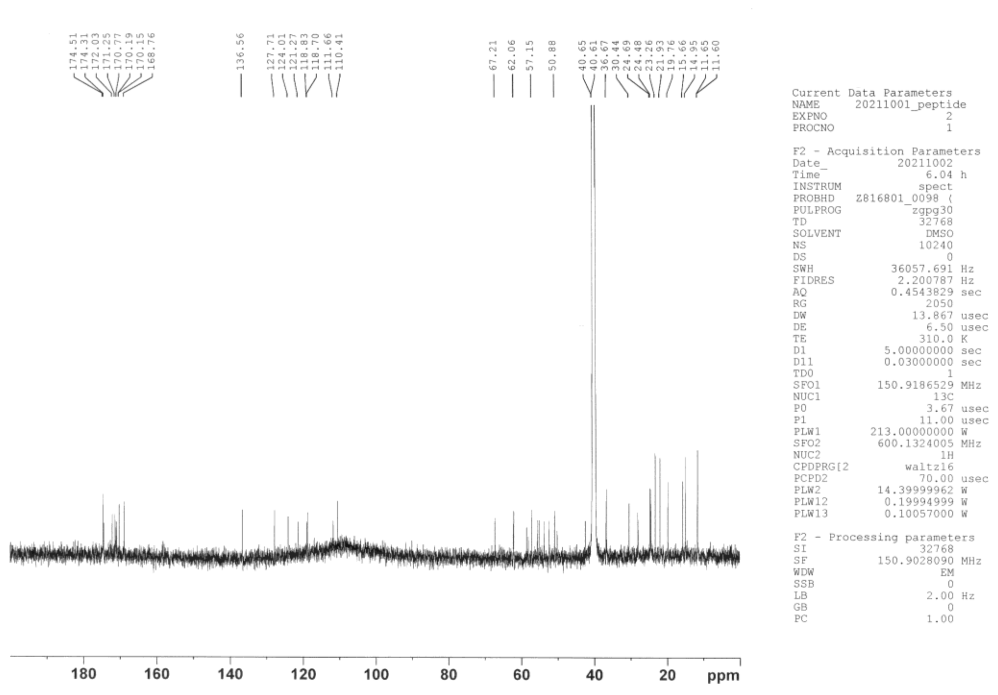

Supplementary Figure 15-2 ^{13}C NMR spectrum of peptide 1.

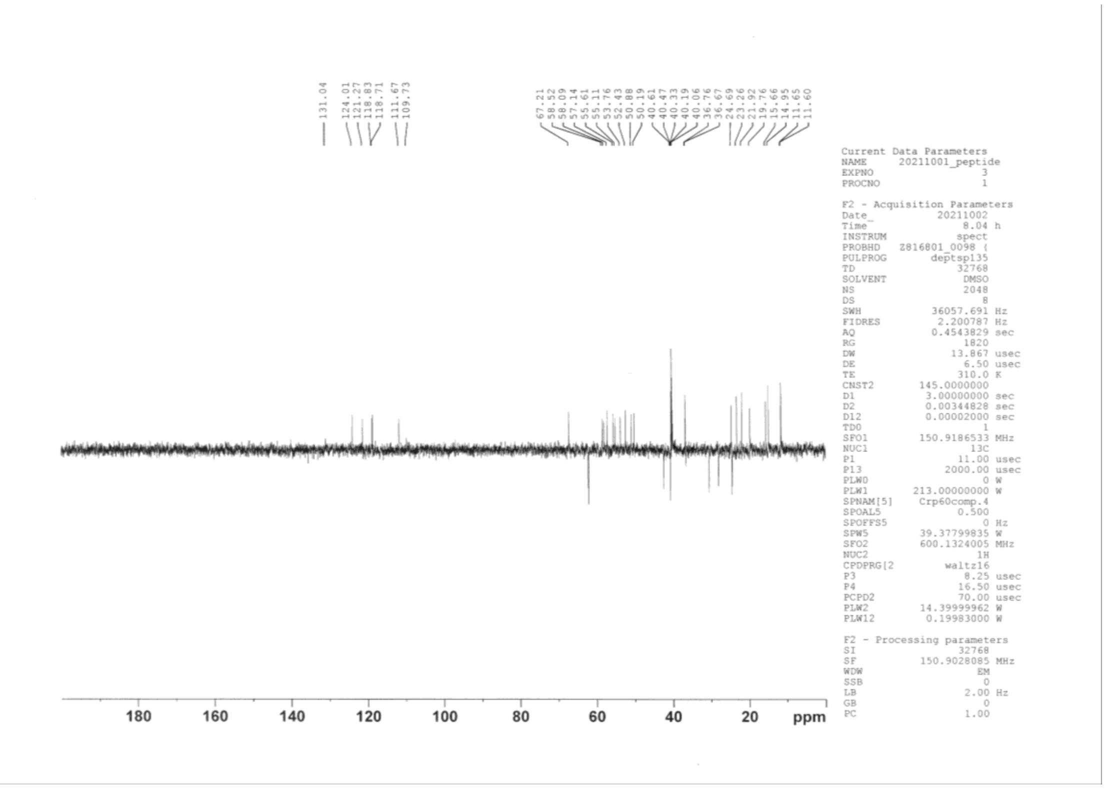

Supplementary Figure 15-3 DEPT135 NMR spectrum of peptide 1.

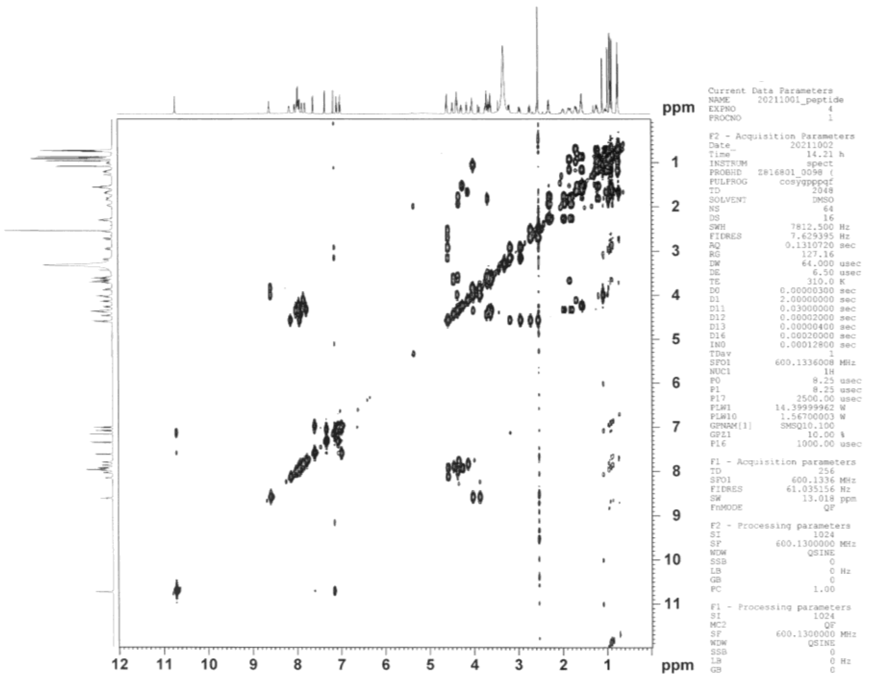

Supplementary Figure 15-4 COSY NMR spectrum of peptide 1.

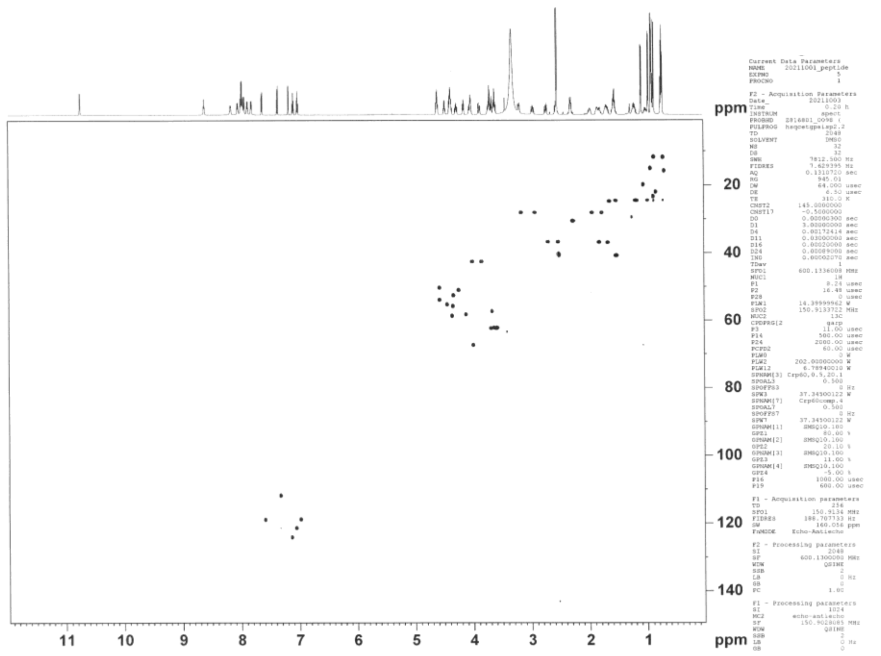

Supplementary Figure 15-5 HSQC NMR spectrum of peptide 1.

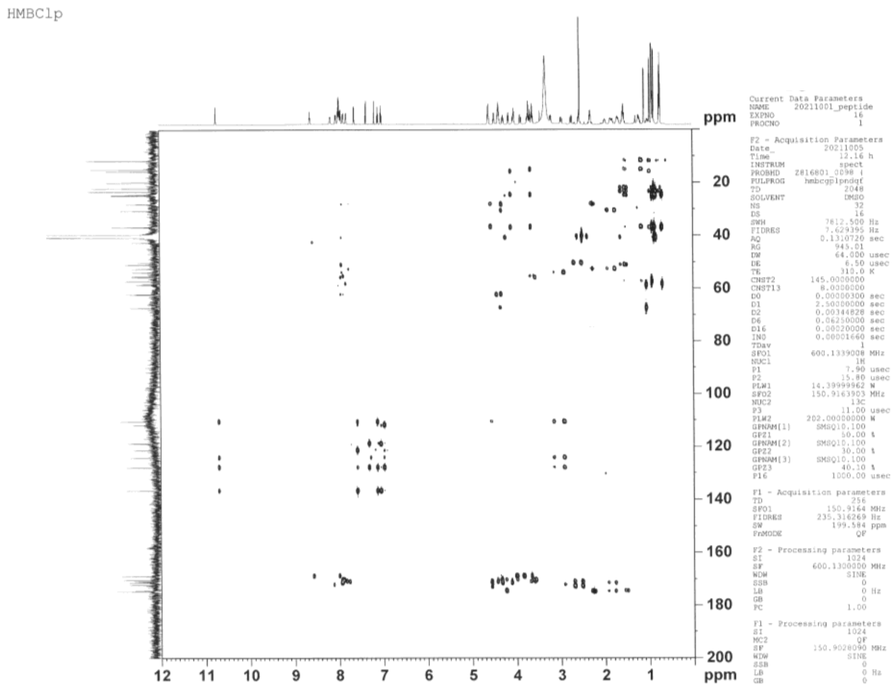

Supplementary Figure 15-6 HMBC NMR spectrum of peptide 1.

.

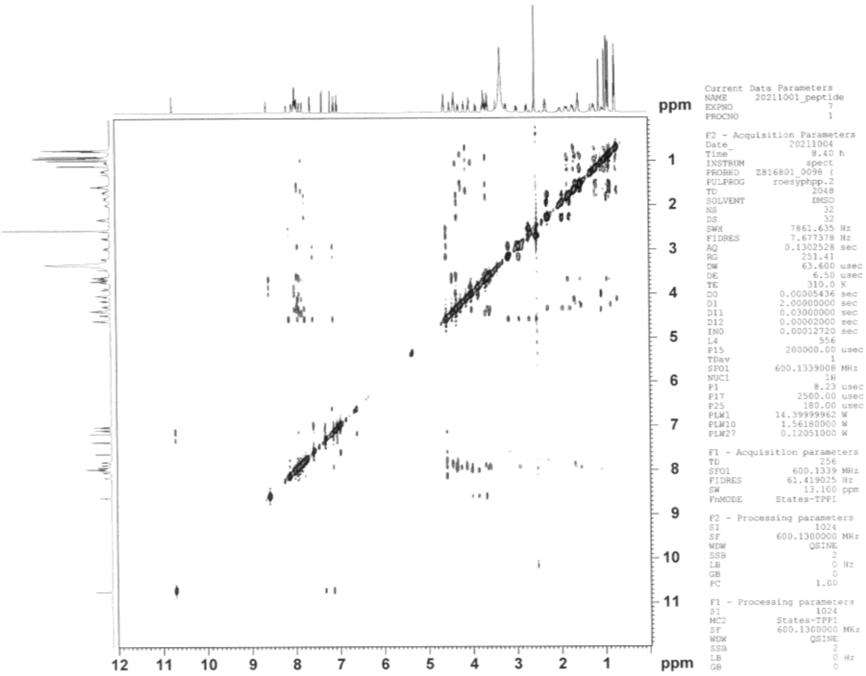

Supplementary Figure 15-7 ROESY NMR spectrum of peptide 1.

TOOLS Peptide (GTR-1 positive2)

Cat. no. TCP-95-10mg

Storage: Store the lyophilized powder at 2–8 °C. After reconstitution, aliquot and store at –20 °C. Repeated freezing and thawing is not recommended.

Peptide sequence:

IGTSIWDESL

Purity and size:

96.72% ,10mg

Notes

This product is for R&D use only, not for drug, household, or other uses. Please consult the Material Safety Data Sheet for information regarding hazards and safe handling practices.

MS Spectrum

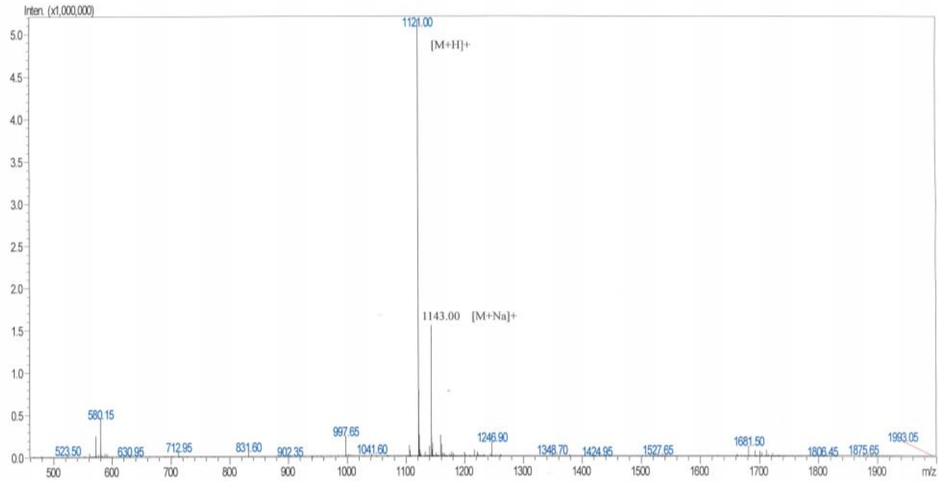

Acquired by :Shen
Data Acquired : 2020/7/19
Injection Volume : 1
Sample Name : GTR-1 positive2
Mw : 1120.21 1120.21
Lot No. : P200709-VV690102
This sequence is hydrophobic.
Dissolution Conditions: 1mg peptide: 0.4 ml DMSO

Probe	:ESI	Probe bias	:+4.5kv
Nebulizer Gas Floe	:1.5L/min	Detector	:1.2kv
CDL	:-20.0v	T.Flow	:0.2ml/min
CDL Temp	:250℃	B.conc	:50%H2O/50%ACN
Block Temp	:400℃		

Supplementary Figure 16 The peptide 1 synthesis report provided by TOOLS Biotech Company.

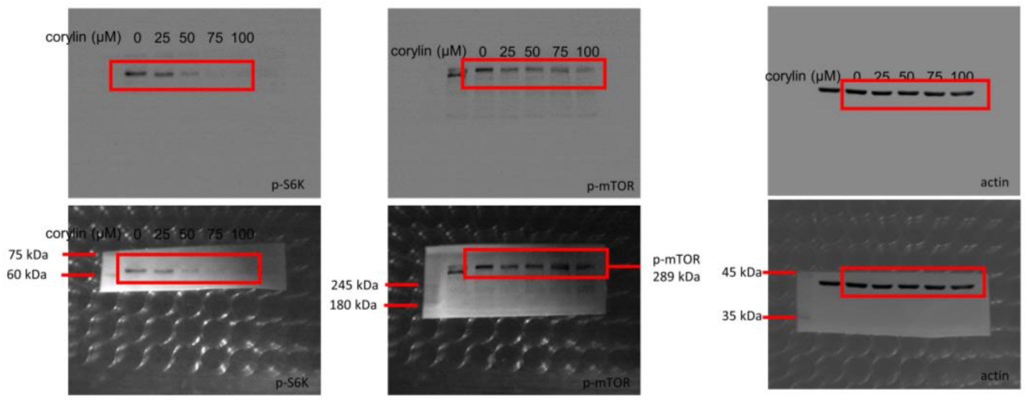

Supplementary Figure 17 The source data of Supplementary Figure 10.
