# Supplementary material for: The flavonoid corylin exhibits lifespan extension properties in mouse
Source: Nat Commun. 2022 Mar 9;13:1238. doi: 10.1038/s41467-022-28908-2 (PMC8907184; doi:10.1038/s41467-022-28908-2)
Supplement: Supplementary file 3 — Reporting Summary [file 41467_2022_28908_MOESM3_ESM.pdf]

nature portfolio

Corresponding author(s): Chin-Chuan Chen

Last updated by author(s): Jan 19, 2022

Reporting Summary

Nature Portfolio wishes to improve the reproducibility of the work that we publish. This form provides structure for consistency and transparency in reporting. For further information on Nature Portfolio policies, see our Editorial Policies and the Editorial Policy Checklist.

Statistics

For all statistical analyses, confirm that the following items are present in the figure legend, table legend, main text, or Methods section.

n/a	Confirmed	
☐	☒	The exact sample size (n) for each experimental group/condition, given as a discrete number and unit of measurement
☐	☒	A statement on whether measurements were taken from distinct samples or whether the same sample was measured repeatedly
☐	☒	The statistical test(s) used AND whether they are one- or two-sided<br>*Only common tests should be described solely by name; describe more complex techniques in the Methods section.*
☒	☐	A description of all covariates tested
☐	☒	A description of any assumptions or corrections, such as tests of normality and adjustment for multiple comparisons
☐	☒	A full description of the statistical parameters including central tendency (e.g. means) or other basic estimates (e.g. regression coefficient) AND variation (e.g. standard deviation) or associated estimates of uncertainty (e.g. confidence intervals)
☐	☒	For null hypothesis testing, the test statistic (e.g. F, t, r) with confidence intervals, effect sizes, degrees of freedom and P value noted<br>*Give P values as exact values whenever suitable.*
☒	☐	For Bayesian analysis, information on the choice of priors and Markov chain Monte Carlo settings
☒	☐	For hierarchical and complex designs, identification of the appropriate level for tests and full reporting of outcomes
☒	☐	Estimates of effect sizes (e.g. Cohen's d, Pearson's r), indicating how they were calculated

Our web collection on statistics for biologists contains articles on many of the points above.

Software and code

Policy information about availability of computer code

Data collection	western blot images were acquired from Wealtec KETA-CL imaging system magic-1D. Cell images were captured using PCO camware64. fluorescent microscopy images were captured using Nikon NIS-Element BR4.0. Molecular docking was performed by BIOVIA Discovery Studio v19.1.0.18287.
Data analysis	OpenCFU 3.8 was used for colony analysis. GraphPad Prism (6.0) was used for graphic visualization and statistical analysis.

For manuscripts utilizing custom algorithms or software that are central to the research but not yet described in published literature, software must be made available to editors and reviewers. We strongly encourage code deposition in a community repository (e.g. GitHub). See the Nature Portfolio guidelines for submitting code & software for further information.

Data

Policy information about availability of data

All manuscripts must include a data availability statement. This statement should provide the following information, where applicable:

- Accession codes, unique identifiers, or web links for publicly available datasets
- A description of any restrictions on data availability
- For clinical datasets or third party data, please ensure that the statement adheres to our policy

The data generated in this study are provided in the Supplementary Information/Source Data files. Source data are provided with this paper. Source data are provided with this paper. Further information will be available from the corresponding author on reasonable request (chinchuan@mail.cgu.edu.tw).
The RNA-sequence data used in this study are available in the Harvard Dataverse under accession code 7ERYZN https://dataverse.harvard.edu/dataset.xhtml?persistentId=doi:10.7910/DVN/7ERYZN.

Structure that support the findings of this study was downloaded from the PDB database (https://rcsb.org), with the accession codes 3R7W.

Field-specific reporting

Please select the one below that is the best fit for your research. If you are not sure, read the appropriate sections before making your selection.

☒ Life sciences ☐ Behavioural & social sciences ☐ Ecological, evolutionary & environmental sciences

For a reference copy of the document with all sections, see nature.com/documents/nr-reporting-summary-flat.pdf

Life sciences study design

All studies must disclose on these points even when the disclosure is negative.

Sample size	Sample sizes were chosen according to the standards of the field as well as the basis on previous studies performed.
Data exclusions	No data were excluded from the analyses.
Replication	All experiments were repeated at least in 3 biological studies. All attempts at replication were successful.
Randomization	For animal experiment, the mice were randomly divided into two groups.<br>For in vitro experiments, cells were randomly allocated into control and experimental groups.
Blinding	In this study, experiment outcome are objective and not likely subjected to bias. Quantification of sSA-β-gal staining and MSN translocation were counted manually by different person in our lab.

Reporting for specific materials, systems and methods

We require information from authors about some types of materials, experimental systems and methods used in many studies. Here, indicate whether each material, system or method listed is relevant to your study. If you are not sure if a list item applies to your research, read the appropriate section before selecting a response.

Materials & experimental systems

n/a	Involved in the study	
☐	☒	Antibodies
☐	☒	Eukaryotic cell lines
☒	☐	Palaeontology and archaeology
☐	☒	Animals and other organisms
☒	☐	Human research participants
☒	☐	Clinical data
☒	☐	Dual use research of concern

Methods

n/a	Involved in the study	
☒	☐	ChIP-seq
☒	☐	Flow cytometry
☒	☐	MRI-based neuroimaging

Antibodies

Antibodies used	The following primary antibodies were used for immunoblotting at a 1:1000 dilution: anti-Pgk1 (ab113687; Abcam, UK) and anti-GFP (G1544; Sigma-Aldrich; St. Louis), anti-P21 (#2947; cell signaling, USA), anit- p-mTOR (#2971, cell signaling, USA), anti-T-mTOR (#2972, cell signaling, USA), anti-β-actin for cell (A5441, Sigma-Aldrich, USA), anti-β-actin for mice tissue (GTX109639, GeneTex, USA). Secondary antibodies were obtained from Sigma-Aldrich (St. Louis, MO, USA) and used at a 1:100000 dilutions.
Validation	All commercially available antibodies were validated by manufacturer. We examined primary antibodies to manuals and got similar results with validation result on manufacture website.<br>Pgk1：Species Specificity: Saccharomyces cerevisiae. Tested Applications: WB, ICC/IF<br>GFP：Species Specificity: human, mouse, rat, yeast. Tested Applications: WB, IP<br>P21：Species Specificity: human, monkey. Tested Applications: WB, IP, IHC-P, IF-IC<br>p-mTOR：Species Specificity: human, mouse, rat, monkey. Tested Applications: WB<br>T-mTOR：Species Specificity: human, mouse, rat, monkey. Tested Applications: WB, IP<br>β-actin for cell (A5441, Sigma-Aldrich, USA)：Species Specificity: human, mouse, rat. Tested Applications: WB, IP<br>β-actin for mice tissue (GTX109639, GeneTex, USA)：Species Specificity: human, mouse, rat, monkey. Tested Applications: WB, ICC/IF, IP, IHC-P

Eukaryotic cell lines

Policy information about cell lines

Cell line source(s)	HUVECs were originally obtained from Bioresource Collection and Research Center, Taiwan
Authentication	HUVECs were kindly provided by Professor Shu-Huei Wang were kept in culture using all the standard methods to avoid contamination with other cell line and validated by morphological analysis.
Mycoplasma contamination	HUVECs were tested negative for mycoplasma contamination.
Commonly misidentified lines (See ICLAC register)	NO commonly misidentified lines were used.

Animals and other organisms

Policy information about studies involving animals; ARRIVE guidelines recommended for reporting animal research

Laboratory animals	Yeast: lab strain BY4741; C57BL/6J male mice (34 weeks of age) were used.
Wild animals	This study did not involve wild animals.
Field-collected samples	NO field collected samples were used in this study.
Ethics oversight	C57BL/6J male mice were provided by the National Laboratory Animal Center (NLAC), NAR Labs, TaiwanAll animal procedures were approved by Chang Guan University Animal Care Center (IACUC protocol no. CGU15-150).No IRB or IACUC approval is required for using laboratory yeast and cells.

Note that full information on the approval of the study protocol must also be provided in the manuscript.
